# Metabolic Characterization of the Anthocyanidin Reductase Pathway Involved in the Biosynthesis of Flavan-3-ols in Elite Shuchazao Tea (*Camellia sinensis*) Cultivar in the Field

**DOI:** 10.3390/molecules22122241

**Published:** 2017-12-15

**Authors:** Lei Zhao, Xiao-Lan Jiang, Yu-Mei Qian, Pei-Qiang Wang, De-Yu Xie, Li-Ping Gao, Tao Xia

**Affiliations:** 1 State Key Laboratory of Tea Plant Biology and Utilization, Anhui Agricultural University, 130 West Changjiang Rd., Hefei 230036, China; zhaolei_tea@163.com (L.Z.); jiangxiaolan128@163.com (X.-L.J.); qianym306@126.com (Y.-M.Q.); wpqtea@163.com (P.-Q.W.); 2 College of Horticulture, Qingdao Key Laboratory of Genetic Improvement and Breeding in Horticultural Plants, Qingdao Agricultural University, 700 Changcheng Rd., Qingdao 266109, China; 3 Department of Plant and Microbial Biology, North Carolina State University, Raleigh, NC 27695, USA; 4 School of Life Science, Anhui Agricultural University, 130 West Changjiang Rd., Hefei 230036, China

**Keywords:** anthocyanidin reductase, (+)-catechin, (−)-epicatechin, (−)-epicatechin-gallate, (−)-epigallocatechin, (−)-epigallocatechin-gallate, (+)-gallocatechin

## Abstract

Anthocyanidin reductase (ANR) is a key enzyme in the ANR biosynthetic pathway of flavan-3-ols and proanthocyanidins (PAs) in plants. Herein, we report characterization of the ANR pathway of flavan-3-ols in Shuchazao tea (*Camellia sinesis*), which is an elite and widely grown cultivar in China and is rich in flavan-3-ols providing with high nutritional value to human health. In our study, metabolic profiling was preformed to identify two conjugates and four aglycones of flavan-3-ols: (−)-epigallocatechin-gallate [(−)-EGCG], (−)-epicatechin-gallate [(−)-ECG], (−)-epigallocatechin [(−)-EGC], (−)-epicatechin [(−)-EC], (+)-catechin [(+)-Ca], and (+)-gallocatechin [(+)-GC], of which (−)-EGCG, (−)-ECG, (−)-EGC, and (−)-EC accounted for 70–85% of total flavan-3-ols in different tissues. Crude ANR enzyme was extracted from young leaves. Enzymatic assays showed that crude ANR extracts catalyzed cyanidin and delphinidin to (−)-EC and (−)-Ca and (−)-EGC and (−)-GC, respectively, in which (−)-EC and (−)-EGC were major products. Moreover, two ANR cDNAs were cloned from leaves, namely CssANRa and CssANRb. His-Tag fused recombinant CssANRa and CssANRb converted cyanidin and delphinidin to (−)-EC and (−)-Ca and (−)-EGC and (−)-GC, respectively. In addition, (+)-EC was observed from the catalysis of recombinant CssANRa and CssANRb. Further overexpression of the two genes in tobacco led to the formation of PAs in flowers and the reduction of anthocyanins. Taken together, these data indicate that the majority of leaf flavan-3-ols in Shuchazao’s leaves were produced from the ANR pathway.

## 1. Introduction

It is estimated that more than 2 billion people drink tea every day. More than 50 countries cultivates tea [*Camellia sinensis* (L.) O. Kuntze] plants. China has cultivated tea for more than 2000 years. During the long cultivation history, numerous elite tea varieties have been cultivated for health benefits. To date, approximately 246 cultivars, such as Longjing and Shuchaozao, are grown for multiple nutraceutical benefits and aroma flavors [1]. Based on reports from Chinese Government, approximately 9.78 million tons of fresh leaves were harvested from different cultivars to produce various tea products in 2016, and the economic value was approximately $25.6 billion. Many tea products from China are sold to different regions all over the world.

A daily basis of drinking tea benefits human health. This is because tea contains high contents of polyphenols and other nutraceuticals. The primary tea polyphenols are composed of flavones, flavonols, flavan-3-ols, anthocyanins, proanthocyanidins (PAs), and other specialized forms of flavonoids, among which flavan-3-ols (catechins) account for 70–80% of the total content [2]. Flavan-3-ols and PAs are the main astringent metabolites in tea beverages. Numerous studies have demonstrated that flavan-3-ols possess multiple pharmaceutical functions, such as antioxidative [3], antimutagenic, anticarcinogenic [4], antidiabetic [5], antibacterial, anti-inflammatory, antihypertensive, and anticardiovascular disease activities [6], UV-B protection, body weight control [7], and prevention against Parkinson’s disease and other aging diseases [8].

**Figure 1:**
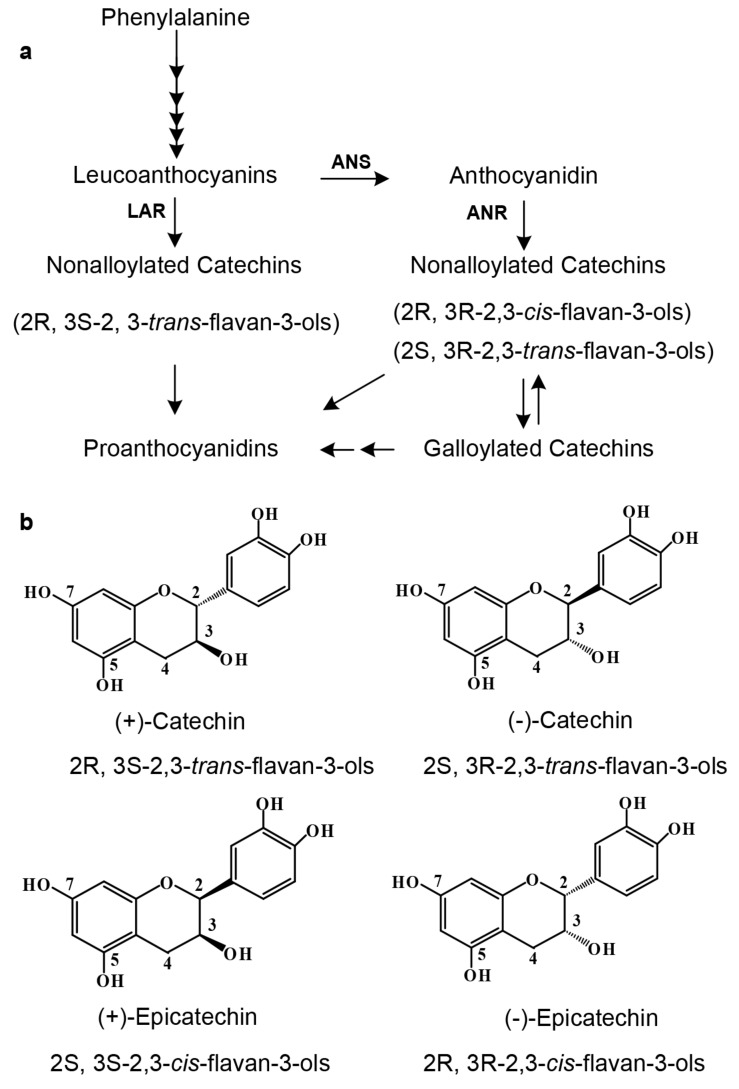
Biosynthetic pathways of flavan-3-ols and proanthocyanidins starting from leucoanthocyanidins in tea plant. (**a**) the ANR pathway to 2*R*,3*R*-2,3-*cis*-flavan-3-ols and 2*S*,3*R*-2,3-*trans*-flavan-3-ols via anthocyanidins and the LAR pathway to 2*R*,3*S*-2,3-*trans*-flavan-3-ols; (**b**) four stereo structures of catechin. LAR, leucoanthocyanidin reductase; ANS, anthocyanidin synthase; ANR, anthocyanidin reductase.

The main composition of tea flavan-3-ols has been appropriately characterized by numerous investigations. Primary aglycones of flavan-3-ols include (+)-catechin [(+)-Ca)] and (−)-epicatechin [(−)-EC)] (Figure 1b), and (+)-gallocatechin [(+)-GC)] and (−)-epigallocatechin [(−)-EGC)] [9]. Primary conjugates are composed of (−)-epciatechin-3-*O*-gallate [(−)-ECG)] and (−)-epigallocatechin-3-*O*-gallate [(−)-EGCG)]. The formation of (−)-ECG and (−)-EGCG was recently characterized by enzyme investigations. Two enzymes, namely UDP-glucose: galloyl-1-*O*-β-d-glucosyltransferase (UGGT) and 1-*O*-galloyl-β-d-glucose *O*-galloyltransferase (ECGT) [2], were isolated from tea leaves. These two enzymes were demonstrated to add gallic acid at the 3-*O* positon of both (−)-EC and (−)-EGC to form (−)-ECG and (−)-EGCG.

Since 2003, two biosynthetic pathways have been characterized to control the formation of flavan-3-ols, each with different stereo-configuration preference (Figure 1a) [9,10,11,12]. One is the anthocyanidin reductase (ANR) pathway, which starts with leucoanthocyanidins and then undergoes anthocyanidins to three stereo types of flavan-3-ols, such as (−)-Ca, (−)-EC, and (+)-EC. This pathway has been shown to exist in the plant kingdom across ferns, gymnosperms, and angiosperms [13]. This pathway has also been used to develop metabolic engineering technology for production of PAs in commercial crops, such as tobacco plant [14,15] and forage crops (such as *Medicago trucatula* and *M. sativa*) [14,16]. The other is the leucoanthocyanidin reductase (LAR) pathway, which is from leucoanthocyanidins to one stereo type of flavan-3-ols, such as (+)-Ca (Figure 1b) [13]. Although *Arabidopsis thaliana* does not contain the LAR pathway [10], multiple plants have been shown to include it [13]. To date, both the ANR and LAR pathways have been demonstrated to associate with the formation of PAs in one tea cultivar [17,18]. Two *ANR* homologs, namely *CsANR1* and *CsANR2*, were recently cloned from a blister blight-resistant tea cultivar TRI2043 grown in Sri Lanka [17]. Unlike ANRs from *A. thaliana* and *M. trucatula*, which catalyze cyanidin to (−)-EC and (−)-Ca, recombinant CsANR1 and CsANR2 were shown to catalyze this substrate to (+)-EC and (+)-Ca [17]. In addition, the ectopic expression of *CsANR1* and *CsANR2* in tobacco plants were reported to lead to the formation of PAs in anthocyanin producing tissues. One LAR, namely *CsLAR*, was also cloned and functionally characterized from this tea cultivar. On the one hand, the recombinant CsLAR was characterized to convert leucocyanidin to (+)-Ca. On the other hand, the ectopic expression of *CsLAR* led to the formation of (−)-EC in anthocyanin producing tissues of tobacco plants [17].

Shuchazao is an elite cultivar. It produces orchid-like aroma. To date, it is one of primary cultivars grown in Anhui and Henan provinces, China. The total cultivation field is around 14,000 hectares. The annual fresh leaf yield is 100,000–140,000 tons to produce different superior green tea products [Data from Tea Research Institute of China Academy of Agriculture Sciences AS (TRI, CAAS)] (http://www.e-chinatea.cn/other_shujuku.aspx). Here, we report to characterize the ANR pathway in Shuchazao grown in the field. LC-MS based metabolic profiling identified six flavan-3-ol molecules including (+)-Ca, (−)-EC, (+)-GC, (−)-EGC, (−)-ECG, and (−)-EGCG. The contents of these six molecules were different in buds and four positional leaves, which are usually used as primary materials to produce tea products. Enzyme analysis showed that crude protein of fresh leaves converted cyanidin and delphinidin to (−)-EC and (−)-EGC as major products and (−)-Ca and (−)-GC as minor products, respectively. Two cDNAs, namely *CssANRa* and *CssANRb*, were cloned from young leaf tissues and expressed in *E. coli* to induce recombinant proteins. Enzymatic analysis demonstrated that CssANRa catalyzed anthocyanidins to 2*S*,3*R*-2,3-*trans*, 2*R*,3*R*-2,3-*cis*-flavan-3-ols, and 2*S*,3*S*-2,3-*cis*-flavan-3-ols such as (−)-Ca, (−)-EC and (+)-EC. CssANRb converted anthocyanidins to 2*S*,3*R*-2,3-*trans* and 2*S*,3*S*-2,3-*cis*-flavan-3-ols such as (−)-Ca and (+)-EC. Transgenic analysis further demonstrated that the overexpression of either of the two genes led to the formation of PAs in transgenic tissues. These data are informative to regulate flavan-3-ol biosynthesis in this elite tea for producing high quality tea products.

## 2. Results

### 2.1. Flavan-3-ol Profile in New Leaves

**Figure 2:**
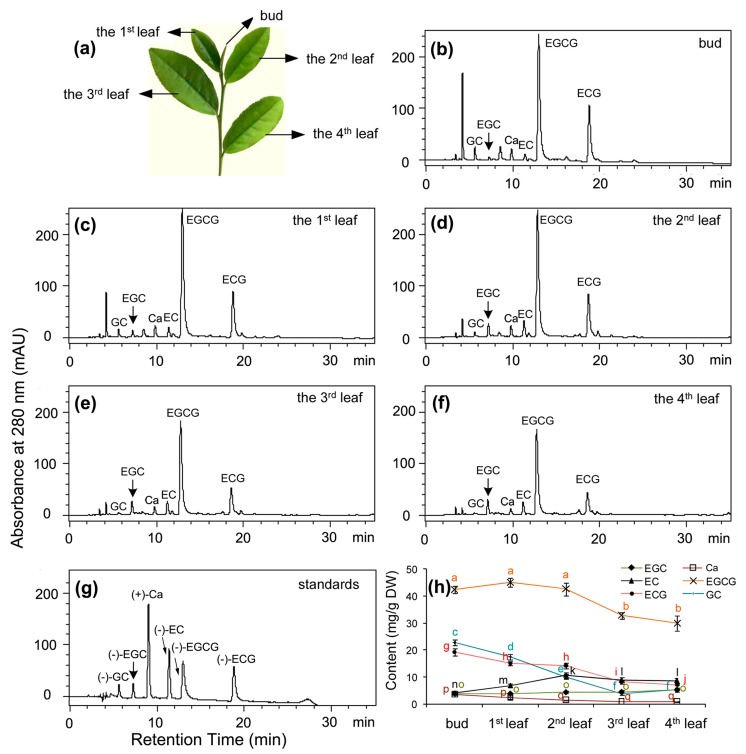
Flavan-3-ol profiles in leaves of 10-year-old Shuchazao plants grown in the field. (**a**) leaf morphologies on a new branch in spring; (**b**–**e**) HPLC profiles showing flavan-3-ols from buds (**b**), the 1st leaf (**c**), the 2nd leaf (**d**), the 3rd leaf (**e**), and 4th leaf (**f**). (**g**) standards, (+)-Ca: catechin, (−)-EC: (−)-epicatechin, (−)-GC: (−)-gallocatechin, (−)-EGC: (−)-epigallocatechin, (−)-ECG: (−)-epicatechin-3-gallate, and (−)-EGCG: (−)-epigallocatechin-3-gallate; (**h**) contents of six metabolites in leaves and buds (on each colored line, points labeled with different low case letters mean significant difference, *p*-value less than 0.05, while labeled with the same ones mean insignificance, *p*-value higher than 0.05. *p*-Values were calculated with Student’s *t*-test).

In tea industry, buds and leaves at the top four positions, which are developed on new branches of Shuchazao shrub (Figure 2a) in the early spring, form primary materials to produce different quality tea products. Accordingly, new leaves at these positions were collected to characterize flavan-3-ol profiles. Based on authentic standards, four flavan-3-ol aglycones and two galloylated conjugates, which are (+)-catechin [(+)-Ca], (−)-epicatechin[(−)-EC], (+)-gallocatechin[(+)-GC], (−)-epigallocatechin [(−)-EGC], (−)-epicatechin-3-gallate [(−)-ECG], and (−)-epigallocatechin-3-gallate [(−)-EGCG] (Figure 2b–f), were identified in extracts of five positional samples.

The contents of these six metabolites was calculated based on their standard curves. The resulting data showed that in regardless of positions, the content of (−)-EGCG was the highest, from 3.0% to 4.5% (g/g, DW in five positional leaves. By contrast, the content of (+)-Ca was the lowest, from 0.1–0.3% in five position leaves. The contents of (−)-ECG, (+)-GC, (−)-EC, and (−)-EGC were in a range of 0.9–1.9%, 0.4–2.3%, 0.4–1.1%, and 0.4–0.5% (g/g, DW) in the five positional samples, respectively.

Among the five positional samples, the buds contained the highest total contents of flavan-3-ols, approximately 9.6% (g/g, DW). The 4th position leaves contained the lowest total content, approximately 5.7% (g/g, DW). Among six metabolites, except for (−)-EGC and (−)-EC, four metabolites showed an apparent decrease trend from the 2nd leaf to the 4th leaf. This result provides evidence indicating that different quality of tea products is associated with these sample positions.

### 2.2. ANR Activity in New Leaf Samples

**Figure 3:**
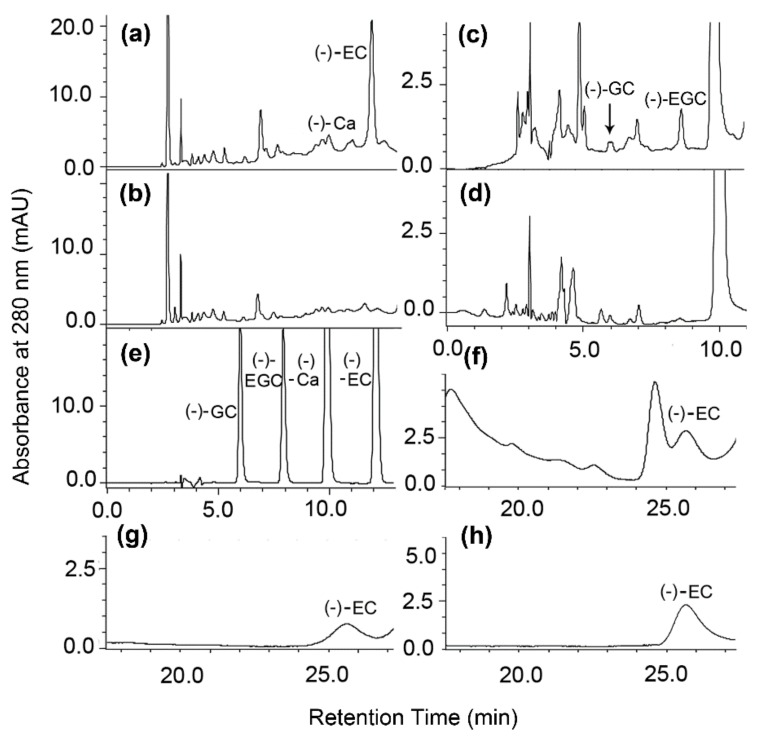
Anthocyanidin reductase (ANR) activity analysis using crude protein extracts from pooled fresh leaves. (**a**,**b**) HPLC profiles show the formation of (−)-catechin [(−)-Ca] and (−)-epicatechin [(−)-EC] produced from incubations of cyanidin with crude ANR extracts (**a**) but not boiled ones (**b**). (**c**,**d**) HPLC profiles show the formation of (−)-gallocatechin [(−)-GC] and (−)-epigallocatechin [(−)-ECG] produced from incubations of delphinidin with crude ANR extracts (**c**) but not from boiled ones (**d**). (**e**) four positive standards separated with the same conditions used in (**a**–**d**). (**f**,**g,h**) normal phase chiral column based profiles of (−)-EC from incubation of crude ANR extract with cyanidin (**f**), purified (−)-EC (**g**), and (−)-EC standard (**h**).

Crude enzyme analysis has been reported being an effective method to demonstrate the ANR pathway in plants [13]. To understand whether the formation of the six flavan-3-ol molecules is associated with an ANR activity, we pooled new leaves from the five positions (Figure 2a) to extract crude enzyme. Based on available authentic standards, retention time, and UV-spectrum properties, HPLC analysis showed that the incubation of crude ANR extract and cyanidin resulted in two new metabolites, (−)-EC and (−)-Ca (Figure 3a). In addition, (−)-GC and (−)-EGC were formed in the incubation of crude ANR extract and delphinidin (Figure 3c). However, these metabolites were not observed in control incubations (Figure 3b,d), supporting that the four metabolites resulted from the ANR catalysis. Furthermore, HPLC profiles showed that both (−)-EC and (−)-EGC formed major enzymatic products (Figure 3a,c), supporting the metabolic profile that (−)-EGCG, (−)-ECG, (−)-EC, and (−)-EGC were four primary metabolites in leaves collected from field grown plants.

To further confirm the chirality of enzymatic products separated by reverse phase column, the major product derived from the incubation of crude ANR extract and cyanidin was isolated on TLC plates according to our previous reports [9]. Based on the (−)-EC standard, analysis of HPLC coupled with a normal phase-chiral column identified that this metabolite was (−)-EC (Figure 3f,h). However, (+)-EC was not found from enzyme reaction products. This result supported that the 2*R*,3*R*-2,3-*cis* stereo types, such as (−)-EC and (−)-EGC, accounted for most of flavan-3-ol metabolites synthesized in leaves described above.

### 2.3. Field-Grown Shuchazao Leaves Express Two ANR Homologs

We previously submitted GenBank two *ANR* sequences (GenBank: KY615701.1 and GenBank: KY615702.1), which have been curated by NCBI. Based on these two sequences, we isolated two *ANR* cDNA sequences from new leaves of Shuchazao. In addition to ours reported here, two *ANR* homologs, namely *CsANR1* and *CsANR2*, were reported from the blister blight-resistant tea cultivar TRI2043 in Sri Lanka [17]. To distinguish from these two terms, we named ours as *CssANRa* and *CssANRb*, in which *Css* resulted from abbreviation of *Camelina sinensis* cv. Shuchazao.

**Figure 4:**
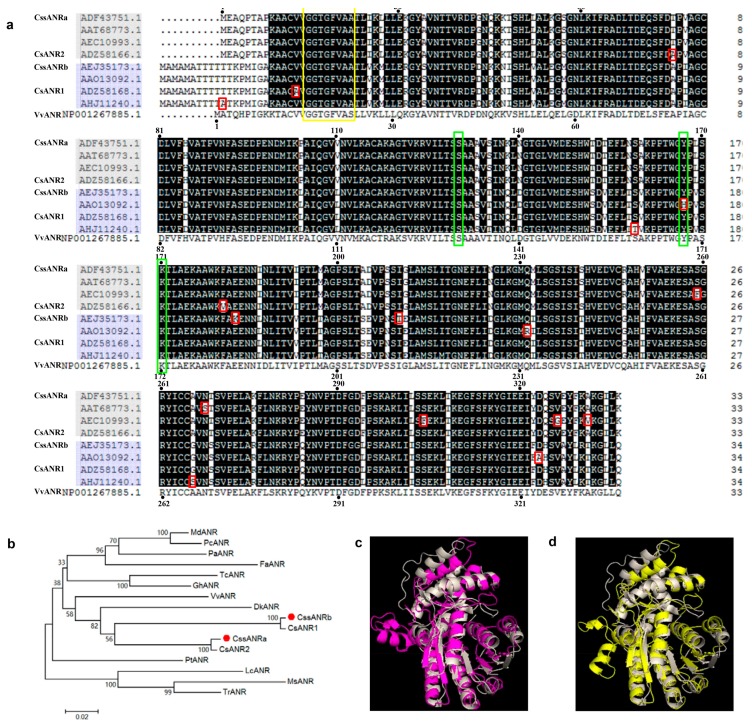
Molecular characterization of two *ANR* homologs. (**a**) A sequence alignment shows both difference and identity of amino acids in nine homologs. Those different amino acids are highlighted using a small red rectangle. The yellow frame shows G-rich NADPH and NADH binding domain; (**b**) a phylogenetic tree established using 16 ANR homolog amino acid sequences; (**c**,**d**) Three dimensional structure prediction for CssANRa (**c**) and CssANRb (**d**) using grape ANR template (2rh8A), gray: 2rh8A, red: CssANRa and yellow: CssANRb.

To date, in addition to two *ANR* cDNAs from TRI2043 as well as *CssANRa* and *CssANRb* reported here, four additional *ANR* cDNA sequences from other tea cultivars are currently curated at the National Center for Biotechnology Information (NCBI). Their GenBank accession numbers are KY615701.1, KY615702.1, HM003282.1, GU992402.1, KF879515.1, and AY169404.1. In addition, the corresponding GenBank accession numbers for amino acid sequences of these four cDNAs are ASU87432.1, ASU87433.1, ADZ58166.1, AAO13092.1, ADZ58168.1, and AHJ11240.1. Amino acid sequences deduced from eight tea ANR cDNAs including ours were aligned to compare their sequence identity and difference. In addition, a grape (*Vitis vinifera*) ANR homolog, VvANR (GenBank: CAD91911), was used as reference for alignment. The sequence length of VvANR includes 337 amino acids. The aligning result revealed a feature of two groups of tea *ANR* sequences (Figure 4a), each of which was composed of four sequences. It is interesting that four members in the first group are composed of 336 amino acids, 10 shorter in the N-terminus than the four members in the second group consisting of 346 ones. In addition, the two groups have 51 additional different amino acid residues, 47 of which are completely conserved in the first group, while 48 of which are conserved in the second group, respectively (Figure 4a). In addition to these interesting differences, a few of scattered amino acid residue differences were observed in eight sequences (Figure 4a). Sequences of the G-rich NADPH and NADH binding domain and three catalytic amino acid residues (Ser, Tyr, and Lys) are conserved in all homologs (Figure 4a).

To understand the phylogenetic relationship, amino acid sequences of four tea ANR homologs (CssANRa, CsANR1, CssANRb, and CsANR2) and 12 additional other plant ANR homologs were used to build a tree. The resulting unrooted tree showed that four tea ANR homologs, VvANR, and DkANR were grouped in the same clade (Figure 4b).

Based on the reported crystal structure (2rh8A) of VvANR [19], potential three-dimensional structures for CssANRa and CssANRb were predicted using the Swiss Model software (http://swissmodel.expasy.org/). The resulting models characterized structural similarity between VvANR and CssANRa and CssANRb (Figure 4c,d). Based on the crystal structure of 2rh8A, CssANRa and 2 have the conservative NAD(P)H binding domain (Gly16-Gly17-Thr18-Gly19-Phe20-Val21-Ala22-Ser23) (Figure 4a, framed by yellow color) and catalytic sites (Ser131, Tyr168, and Lys172 for VvANR (Figure 4a, framed by green color).

### 2.4. Expression Profiles of CssANRa and CssANRb

**Figure 5:**
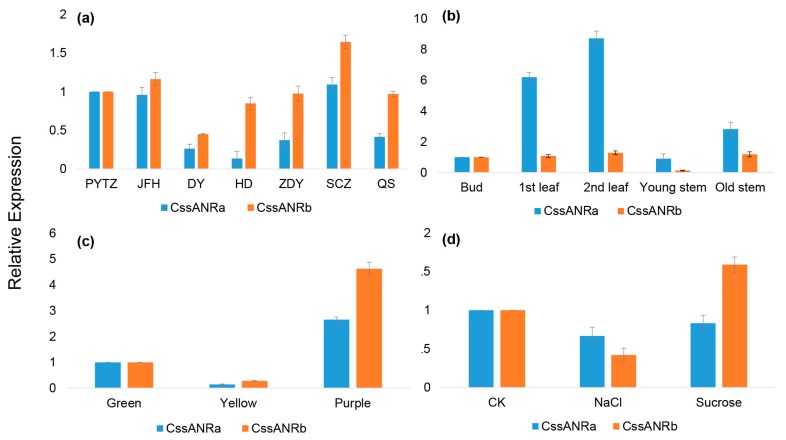
Expression patterns of *CssANRa* and *CssANRb*. (**a**) Relative expression levels of *CssANRa* and *CssANRb* in the 2nd leaf from seven different tea varieties, PYTZ (*C. sinensis* cv. Pingyangtezao), JFH (*C. sinensis* cv. Jinfenghuang), DY (*C. sinensis* cv. Dayewulong), HD (*C. sinensis* cv. Huangdan), ZDY (*C. sinensis* cv. Zhengdayin), SCZ (*C. sinensis* cv. Shuchazao), QS (*C. sinensis* cv. Queshe); (**b**) Relative expression levels of *CssANRa* and *CssANRb* in different tissues (bud, the 1st leaf, the 2nd leaf, young stem, and old stem) of Shuchazao; (**c**) Relative expression levels of *CssANRa* and *CssANRb* in three types of pigmented leaves characterized by green, yellow and purple leaves. Each type of pigmented leaf sample was collected a group of plants. The green group includes *C. sinensis* cv. Shuchazao, *C. sinensis* cv. LongJing, and *C. sinensis* cv. Tieguanyin. The yellow group consists of *C. sinensis* cv. Huangjinya, *C. sinensis* cv. Zhonghuangyihao, *C. sinensis* cv. Zhonghuangerhao. The purple group is composed of *C. sinensis* cv. Zijuan, *C. sinensis* cv. Ziyan, and *C. sinensis* cv. Sunrouge. Leaf samples in each group were collected from their three varieties and then pooled together for gene expression analysis; (**d**) Relative expression levels of *CssANRa* and *CssANRb* in Shuchazao leaves treated by 200 mM NaCl solution 4 h or 200 mM sucrose solution 4 h.

Quantitative RT-PCR was carried out to characterize expression profiles of *CssANRa* and *CssANRb* in seven tea varieties, five types of tissues, three types of pigmented leaves, and two types of treatments. The resulting data showed that *CssANRa* and *CssANRb* had different expression levels among seven varieties. Their expression levels were high in Shuchazao, Pingyangtezao, and Jinfenghuang, but low in Huangdan and Dayewulong (Figure 5a). The expression pattern of *CssANRa* and *CssANRb* in five tissues of Suchazao from the highest to the lowest levels were in the order of the 2nd, 1st leaves, old stems, bud, and young stems (Figure 5b). In three types of pigmented leaves, their expression levels were the highest in purple leaves, followed by green, and then yellow leaves (Figure 5c). The expression levels of *CssANRa* was reduced by both NaCl and sucrose treatments, while the expression levels of *CssANRb* was decreased by NaCl treatment but increased by sucrose treatment (Figure 5d).

### 2.5. Catalytic Activity of Recombinant CssANRa and CssANRb

**Figure 6:**
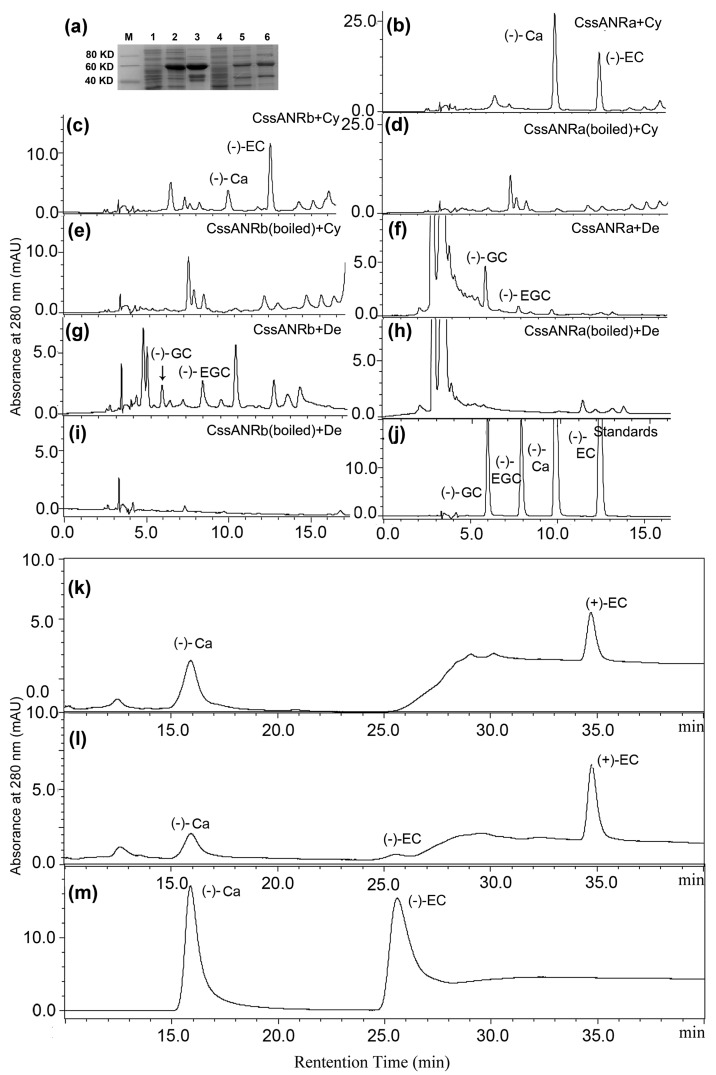
Enzymatic analysis of recombinant CssANRa and CssANRb. (**a**) SDS-PAGE shows induction of recombinant pET31a^+^-CssANRa and T7 SUMO-CssANRb induced in *E. coli*, M: molecular weight markers, 1: protein profile prior to induction of pET31a^+^-CssANRa, 2: protein profile after induction of pET31a^+^-CssANRa for 3 h, 3: partially purification of supernatant of pET31a^+^-CssANRa, 4: prior to induction of T7 SUMO-CssANRb, 5: after induction of T7 SUMO-CssANRb for 3 h, 6: partially purification of supernatant T7 SUMO-CssANRb; (**b**–**e**) HPLC profiles show the formation of (−)-catechin [(−)-Ca] and (−)-epicatechin [(−)-EC] produced from incubations of cyanidin with recombinant CssANRa (**b**) and CssANRb (**c**) but not boiled CssANRa (**d**) and CssANRb (**e**); (**f**–**i**) HPLC profiles show the formation of (−)-gallocatechin [(−)-GC] (**f**) and (−)-epigallocatechin [(−)-EGC] produced from incubations of delphinidin with recombinant CssANRa (**g**) and CssANRb (**h**) but not boiled CssANRa (**i**) and CssANRb (**j**). (**k**), profiles of (−)-Ca, (−)-EC,(−)-GC, and (−)-EGC standards. (**k**–**m**), normal-phase chiral column based separation shows (−)-Ca and (+)-EC from CssANRa (**k**), (−)-Ca, (−)-EC, and (+)-EC from CssANRb (**l**) after incubation with cyanidin, and (−)-Ca and (−)-EC standards (**m**).

The ORFs of *CssANRa* and *CssANRb* were cloned to a pET31a^+^ vector to obtain pET31a^+^-*CssANRa* and -*CssANRb* plasmids to induce recombinant enzymes. After induction with IPTG, *E. coli* harboring the pET31a^+^-*CssANRa* plasmid expressed a soluble recombinant protein, which was shown by SDS-PAGE analysis (Figure 6a). However, no recombinant protein was induced from *E. coli* containing the pET31a^+^-*CssANRb* plasmid. To induce a recombinant *CssANRb*, its ORF was cloned to the T7 SUMO vector to obtain a recombinant plasmid, T7 SUMO-*CssANRb*. After induction with IPTG, *E. coli* harboring this plasmid expressed a recombinant protein (Figure 6a). SDS-PAGE analysis showed that total soluble recombinant protein from each induction contained excessive other proteins. Accordingly, the MagnetHis Protein Purification System (Promega, Madison, WI, USA) was used to reduce those excessive proteins from total crude proteins. SDS-PAGE analysis showed that this treatment obviously reduced certain types of excessive soluble proteins (Figure 6a). Therefore, recombinant CssANRa and CssANRb proteins were partially purified for catalytic analysis.

Recombinant CssANRa and CssANRb were incubated with cyanidin and delphinidin in the presence of NADPH, respectively. HPLC analysis using reverse-phase column separation detected two new peaks at 280 nm from both CssANRa (Figure 6b) and CssANRb assays (Figure 6c) incubated with cyanidin. However, these two new peaks were not detected from boiled enzyme-substrate incubations (Figure 6d,e). These results demonstrated that new peaks were derived from enzymatic catalysis. To characterize these two new peaks, (−)-Ca and (−)-EC standards were used as control (Figure 6j). Based on retention time and UV spectrum, the two new peaks had the same retention time as (−)-Ca and (−)-EC standards, respectively. It was interesting that based on peak area and height values, recombinant CssANRa produced more (−)-Ca than (−)-EC, while recombinant CssANRb produced more (−)-EC than (−)-Ca (Figure 6b,c).

HPLC analysis showed that two new peaks were detected at 280 nm from the incubation of CssANRa and CssANRb with delphinidin (Figure 6f,g), but they were not detected from the boiled proteins (Figure 6h,i). In addition, (−)-GC and (−)-EGC standards were co-eluted as positive controls (Figure 6j). Based on retention time and UV spectrum, these two peaks were (−)-GC and (−)-EGC. 

To characterize the chirality, enzymatic products from cyanidin were analyzed on a normal phase chiral column using HPLC. Both (−)-Ca and (−)-EC were used as positive controls. The resulting chromatographs showed that CssANRa-derived (−)-Ca (Figure 6k) was detected at the same retention time as the standard (Figure 6m). However, an additional peak but not (−)-EC was detected from this separation on the normal phase chiral column (Figure 6k). CssANRb-derived (−)-Ca and (−)-EC (Figure 6l) were detected at the same retention time as (−)-Ca and (−)-EC standards (Figure 6m). In addition, a third metabolite peak was also observed from the CssANRb assay and demonstrated to be (+)-EC (Figure 6l). 

In addition, the optimum temperature values of recombinant CssANRa and CssANRb were 40 °C (Supplementary Figure S1a,b). The optimum pH values of recombinant CssANRa and CssANRb were 6.5 and 5.5, respectively (Supplementary Figure S1c,d).

### 2.6. Overexpression of CssANRa and CssANRb in Tobacco Flowers Reduces Anthocyanins and Produces PAs

**Figure 7:**
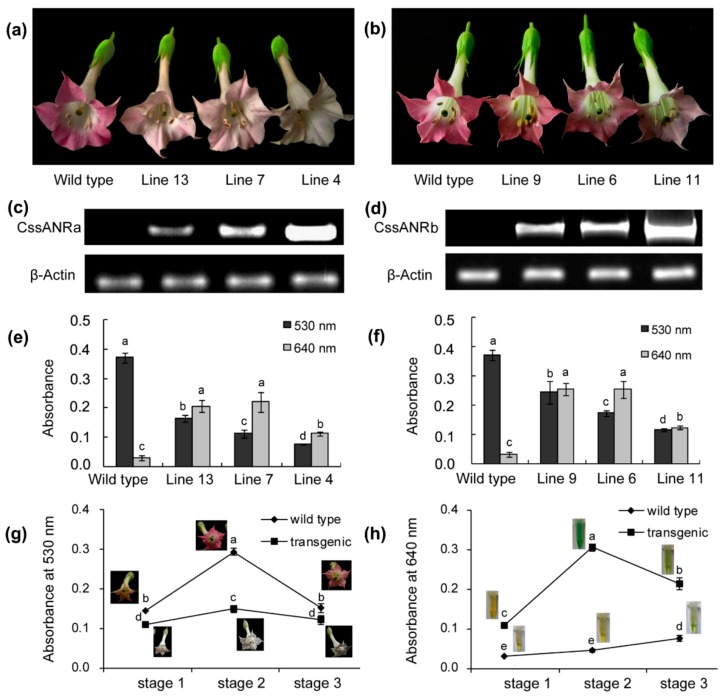
Ectopic expression of *CssANRa* and *CssANRb* genes in tobacco flowers. (**a**,**b**) pigmentation phenotypes of *CssANRa* (**a**, three lines) and *CssANRb* (**b**, three lines) transgenic vs. wild-type tobacco flowers fully opened, (**c**,**d**) gel images showing expression of *CssANRa* (**c**) and *CssANRb* (**d**) transgene fragments amplified from fully opened flowers by semi-quantitative PCR; (**e**) anthocyanin and PA levels in *CssANRa* transgenic vs. wild-type flowers estimated at 530 nm and 640 nm, respectively; (**f**) anthocyanin and PA levels in *CssANRb* transgenic vs. wild-type flowers estimated at 530 nm and 640 nm, respectively; (**g**,**h**) dynamic patterns of anthocyanins (**g**) and PAs (**h**) levels at three selected development stages of *CssANRa* transgenic flowers. In (**e**,**f**), bars labeled with different low case letters for anthocyanin or PAs mean significant difference (*p*-value less than 0.05), while with the same ones mean insignificance (*p*-value higher than 0.05). In (**g**,**h**), points labeled with different low case letters mean significant difference (*p*-value less than 0.05), while with the same ones mean insignificance (*p*-value higher than 0.05). *p*-Values were calculated with Student’s *t*-test.

*CssANRa* and *CssANRb* controlled by a 35S promoter were introduced to tobacco plants, respectively. After selection using antibiotics, 19 and 17 positive transgenic plants were obtained for *CssANRa* and *CssANRb* transgenes. Transgenic and wild-type plants were grown in a glass house to develop flowers, during the growth period of which no phenotypic difference was observed. Flower buds (stage 1, unopened), slightly opening flowers (stage 2), and fully opening flowers (stage 3) were collected to analyze anthocyanins and PAs. Reduction of red-pigmentation was observed on stage 3 transgenic flowers compared with-type flowers (Figure 7a,b) although effects of the two transgenes on red pigmentation were different. Semi-quantitative RT-PCR analysis performed using fully opened flowers showed that the *CssANRa* and *CssANRb* transgenes were expressed in transgenic flowers but not in wild-type flowers (Figure 7c,d), indicating an association with the reduction of red pigmentation in corolla. Further measurements at 530 nm showed significant reduction of anthocyanin levels in fully opened transgenic flowers of both *CssANRa* and *CssANRb* (Figure 7e,f). In addition, the reaction of DMACA solution with PAs extracted from fully opened transgenic flowers led to blue coloration. Further measurement at 640 nm demonstrated that absorbent values of transgenic flower extracts were significantly higher than those of wild-type flowers (Figure 7e,f). This result indicated that the ectopic expression of each *CssANRa* and *CssANRb* alone led to the formation of PAs.

To further characterize PA formation in transgenic flowers, both anthocyanins and PAs were measured during flower development from unopened buds to fully opened flowers. The resulting data showed that the absorbance of anthocyanins were significantly lower in transgenic flowers than in wild-type flowers at stages 2 and 3 (Figure 7g). The reaction of DMACA and PAs led to blue color. The bluish color resulted from PAs in transgenic flowers was much deeper than that from wild-type flowers. Further measurement at 640 nm showed that the absorbent values were higher from transgenic flowers than wild-type flowers at stages 2 and 3. These results indicated that the increase of PAs was a tradeoff consequence of the decrease of anthocyanins.

## 3. Discussion

Shuchaozao is an elite commercial tea cultivar. The ANR pathway in Shuchaozao leaves for tea products lacks an appropriate characterization, although, to date, this pathway in numerous other plant species has been solidly substantiated in genetics, biochemistry, metabolic engineering, and evolution [12,13,13,19,20], and two *ANR* homologs have also been reported in a blister-resistance tea cultivar. In this study, we focused on characterization of the ANR pathway in field-grown plants’ leaves that are used for commercial tea products. Metabolic profiling revealed that young leaves (Figure 2a) used for tea products were rich in total flavan-3-ols (Figure 2h), which were composed of (−)-EGCG, (−)-ECG, (+)-GC, (−)-EC, (−)-EGC, and (+)-Ca. Of these metabolites, (−)-EGCG accounted for approximately 50% of total flavan-3-ols in five positional leaves although its contents decreased in the fully expanded leaves such as the 3rd and 4th leaves. (−)-EGC accounted for 5–10% of total flavan-3-ols in these leaves. Therefore, (−)-EGC and (−)-EGCG together accounted for 55–60% of total leaf flavan-3-ols. In addition, (−)-EC and (−)-ECG accounted for 25–28% of total flavan-3-ols. As we reported previously, (−)-ECG and (−)-EGCG are derived from galloylation of (−)-EC and (−)-EGC [2], which are only derived from the ANR catalysis. Herein, leaf ANR extracts converted cyanidin and delphinidin to (−)-EC and (−)-Ca, and (−)-EGC and (−)-GC, respectively (Figure 3a–d). Chiral analysis further showed that crude leaf ANR extracts produced (−)-EC but not (+)-EC (Figure 3f–h). Therefore, these data reveal that the ANR pathway in leaves of Shuchaozao is actively responsible for the high total contents of flavan-3-ols. Furthermore, these data indicate that the ANR pathway is closely associated with flavan-3-ols based high quality products. 

To further understand the role of the ANR pathway in this cultivar, gene specific primers were designed to clone full length of cDNAs. Two *ANR* homologs, *CssANRa* and *CssANRb*, were isolated from young leaves. Results from qRT-PCR analysis showed that the expression levels of *CssANRa* were more apparently variable than those of *CssANRb* in different tea varieties and different leaf development stages (Figure 5a,b). By contrast, qRT-PCR analysis characterized that the expression levels of *CssANRb* was more obviously variable than those of *CssANRa* in differently pigmented leaves and in those leaves under NaCl and sucrose treatments (Figure 5c,d). In addition, it was interesting that heterogeneous expression in *E. coli* revealed that *CssANRa* and *CssANRb* exhibited different plasmid preferences to produce recombinant proteins. The pET31a^+^ plasmid was appropriate to induce a recombinant CssANRa but not CssANRb. Instead, the recombinant CssANRb was induced when the pET31 SUMO plasmid was used (Figure 6a). In addition, recombinant CssANRa and CssANRb showed different pH optima. By contrast, the temperature optima of the two recombinant enzymes were the same. As those results obtained from the *CsANR1* and *CsANR2* overexpression reported by Pang et al. [17], the overexpression of *CssANRa* and *CssANRb* also led to the formation of PAs in anthocyanin-producing flowers and reduction of anthocyanins during flower development (Figure 7). These transgenic data provide evidence that *CssANRa* and *CssANRb* are involved in the biosynthesis of PAs.

In our experiments, we observed an interesting different result between leaf ANR and recombinant ANR assays. (+)-EC formed from the recombinant ANR assay (Figure 6k,m) was not observed from the leaf ANR assay (Figure 3f,h). The biochemical mechanism behind this observation is unclear. One possibility might be associated with the difference of native leaf ANR extract and recombinant ANR. Native ANRs were extracted from *Lotus corniculatus*, *Desmodium uncinatum*, *Hordeum vulgare*, *Vitis vinifera*, *Vitis bellula*, *Parthenocissus heterophylla*, *Cerasus serrulata*, and *Dryopteris pycnopteroides* [13]. Enzymatic analysis demonstrated that native ANRs from all of these plant species catalyzed cyanidin to (−)-EC as a major product and (−)-Ca as a minor product. Another interesting possibility may be associated with recombinant vectors. Xie et al. reported that both recombinant AtANR and MtANR fused with pMAL converted cyanidin to (−)-EC and (−)-Ca but no (+)-EC [21]. Although Xie et al. [11] did not use a chiral column in their study, they identified chirality via a dichroism spectrum analysis, which resolved the absolute configurations of flavan-3-ols. It was interesting that (+)-EC was observed in reactions catalyzed by His-tag fused ANR. Gargouri et al. demonstrated that a recombinant *V. vinifera* ANR fused with a His-Tag performed a dual catalytic activity, reduction and epimerization [19]. The recombinant VvANR converted cyanidin to (−)-EC, then to (−)-Ca and (+)-EC. Pang et al. [17] also fused CsANR1 and CsANR2 with a His-Tag, which resulted in the formation of (+)-EC. In our report herein, CssANRa and CssANRb were also fused with His-Tag. These interesting differences indicate that the final understanding of absolute configurations of flavan-3-ols needs more studies of both native plant ANRs and recombinant ANRs.

**Figure 8:**
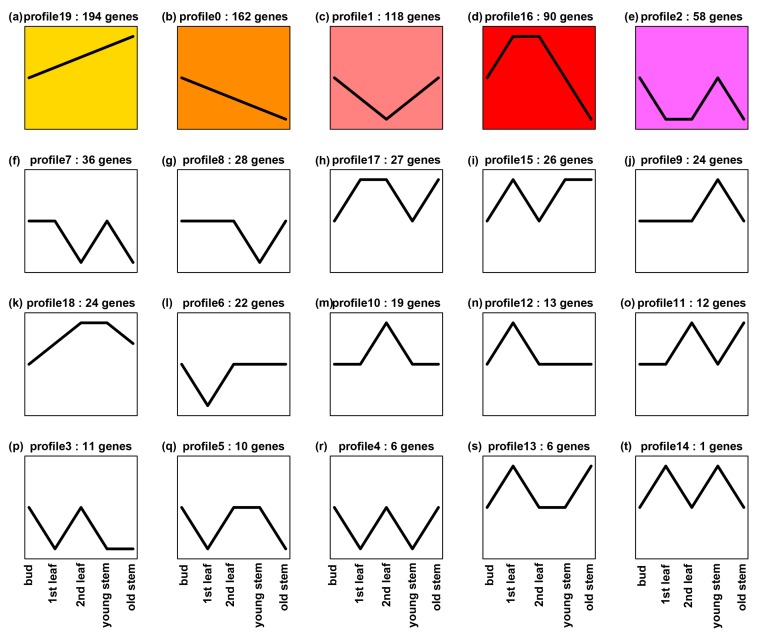
Twenty expression trend profiles established from coupled expression analysis of *ANR* and 885 transcription factor (TF) genes annotated from five transcriptomes sequenced for buds, the 1st leaf, the 2nd leaf, young stem, and old stem. (**a**–**t**) 20 expression trend profiles in 5 tissues. This result reveals that the expression trend of *ANR* is the same as those of 23 TF genes characterized in profile 18 (**k**).

**Figure 9:**
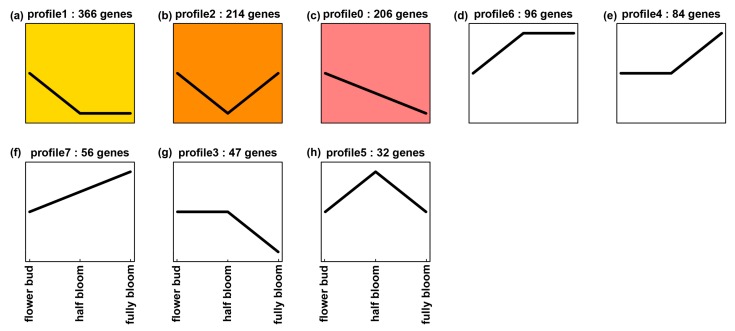
Eight expression trend profiles established from coupled expression analysis of *ANR* and 1110 transcription factor genes annotated from three transcriptomes sequenced for three developmental stage flower samples, flower buds, half blooming (half bloom), and fully blooming (fully bloom). (**a**–**h**) eight expression trend profiles. This result reveals that the expression trend of *ANR* is the same as those of 205 TF genes characterized in profile 0 (**c**).

To enhance general understanding of *ANR* expression in tea tissues, we further mined *ANR* transcripts (without separate two paralogs) in tissues of two other elite green tea varieties, “Pingyangtezao (PYTZ)” and “Ruixue”. Five transcriptomes were sequenced for buds, the 1st leaf, the 2nd leaf, young stem, and old stem of PYTZ, respectively. We obtained transcripts of ANR from all five transcriptomes. In addition, we annotated 1420 potential transcription factor (TF) cDNAs. The transcript of each TF was also obtained from all five transcriptomes. The transcripts of ANR and each TF were used for association analysis of expression profile trend in five tissues. The resulting data revealed the coupled expression of 885 TFs cDNAs and *ANR* in each tissue. A further expression trend analysis using 885 TF genes and *ANR* resulted in 20 expression profiles in buds, the 1st leaf, the 2nd leaf, young stem, and old stem (Figure 8). The transcript trend of *ANR* was the same as those of 23 TF genes, which was characterized in profile 18 (Figure 8k). Further annotation analysis characterized the 23 TFs in 13 families (Supplementary Figure S2), which included four bHLH and 4 MYB members. These data indicate that the expression of *ANR* is positively associated with bHLH and 4 MYB members. Meanwhile, we analyzed gene expression association between *ANR* and TF cDNAs in flowers. Three transcriptomes were sequenced for three developmental stages of flowers of Ruixue. We also obtained transcripts of *ANR* from all three transcriptomes. Moreover, we annotated 1325 TF genes from the three flower transcriptomes and obtained their transcripts in each transcriptome. Based on transcripts of these TF genes and *ANR*, we performed expression association analysis to obtain 1100 TF genes, the expressions of which were coupled with that of *ANR*. Based on transcript of each TF gene and *ANR*, eight expression trend profiles were established (Figure 9). The expression trend of *ANR* was the same as those of 205 TF genes, which was characterized in profile 0 (Figure 9c). The 205 TF genes were annotated to 29 families, which included 74 MYB (MYB family) and 12 bHLH (bHLH family) members (Supplementary Figure S3). These data indicate that the transcript trend of *ANR* is closely associated with those of MYB and bHLH gene members. All of these data will be useful for us to further study the regulation of the ANR pathway in Suchazao and other green tea varieties. 

## 4. Materials and Methods

### 4.1. Field Growth of Shuchazao and Other Varieties for Sampling 

Shuchazao (*Camellia sinensis* (L.) O. Kuntze) plants are grown in an experimental tea garden (research station) at Anhui Agricultural University (latitude 31.86° N, longitude 117.27° E, altitude 20 m above mean sea level) and in the farming field in Hefei, Anhui, China. Plants grown in the tea garden is used for numerous research purposes, while plants grown in the field are used to harvest leaves to produce tea products for sale. Plants were grown 10 years when we performed all experiments reported here. Buds, the 1st leaf, the 2nd leaf, the 3rd leaf, and the 4th leaf (Figure 2a) of new shoots were collected from plants at these two locations. When samples were harvested, they were immediately frozen in liquid nitrogen, transported to laboratory, and then stored in a −80 °C freezer until use for gene cloning and metabolic analysis. In addition, leaves were collected from one year old Shuchazao seedlings and then separated into three groups, which were immediately treated 4 h with 200 mM NaCl and 200 mM sucrose (dissolved in double deionized water) and dd-water as control. Leaf samples treated were immediately frozen in liquid nitrogen and then stored in a −80 °C freezer until RNA isolation and qRT-PCR analysis described below.

In addition, 15 other tea varieties with 10 years old are grown in the same garden for numerous research purposes. Seven were selected to comparatively understand *ANR* gene expression. These are *C. sinensis* cv. Pingyangtezao (PYTZ), *C. sinensis* cv. Jinfenghuang (JFH), *C. sinensis* cv. Dayewulong (DY), *C. sinensis* cv. Huangdan (HD), *C. sinensis* cv. Zhengdayin (ZDY), *C. sinensis* cv. Shuchazao (SCZ), *C. sinensis* cv. Queshe (QS). The 2nd leaf (Figure 2a) was sampled from new shoots of these varieties. Eight other varieties were used to compare *ANR* expression in three different types of pigmented leaves, green, yellow, and purple. The green group included *C. sinensis* cv. LongJing, and *C. sinensis* cv. Tieguanyin. For sample collection, Shuchazao was added to form three varieties in this green group. The yellow group included *C. sinensis* cv. Huangjinya, *C. sinensis* cv. Zhonghuangyihao, and *C. sinensis* cv. Zhonghuangerhao. The purple group consisted of *C. sinensis* cv. Zijuan, *C. sinensis* cv. Ziyan, and *C. sinensis* cv. Sunrouge). The 2nd leaf of new shoots (Figure 2a) was also sampled from each variety in each group for qRT-PCR analysis.

### 4.2. Extraction and Analysis of Flavan-3-ols in Leaves from Field-Grown Shuchazao Plants

Fresh samples, including buds, the 1st leaf, the 2nd leaf, the 3rd leaf, and the 4th leaf (Figure 2a), were grounded into fine powder in liquid nitrogen. A powdered sample (100 mg) was suspended in 1 mL extraction solution (methanol: ddH_2_O, 80:20) in a 1.5 mL tube and sonicated for 10 min at room temperature. After centrifugation at 4000× *g* for 15 min, the resulting upper clear phase was pipetted to a new 1.5 mL tube. The remaining pellet was extracted a second time. Supernatants were combined and the final volume was adjusted to 2 mL. All extracts were filtered through a 0.22 µm membrane for HPLC analysis. 

Extracts from different tissues were analyzed using high performance of liquid chromatography (HPLC) on WATERS600 (Waters, Milford, MA, USA) with a photodiode array detector. Metabolites were separated with a reverse-phase column (Synergi 4u Fusion-RP8 0.5 μm, 250 × 4.6 mm) (Phenomenex, Torrance, CA, USA). Elution solvents were composed of A (1% acetic acid) and B (acetonitrile). A gradient elution program used to separate metabolite was composed of: 0–30 min with 13–30% B, 30–32 min with 13% B, and 32–35 min with 0% B. The flow rate was set 1 mL/min. Ultraviolet (UV) spectra was recorded at 280 nm for identification of flavan-3-ol components. (−)-EC, (+)-Ca, (+)-GC, (−)-EGC, (−)-ECG and (−)-EGCG standards were used as positive controls to identify metabolites from plant tissues and enzyme assays described below.

### 4.3. Extraction of Crude Enzyme from Field-Grown Tea Leaves and Enzyme Assay 

Frozen tea leaves (approximately 25 g, a mixture of the 1st–4th leaves) were ground into fine powder in liquid nitrogen. The powdered sample was completely suspended in 200 mL phosphate buffer (0.1 M, pH 7.4) supplemented with 25 g polyvinyl polypyrrolidone (PVPP) and 5 mM β-mercaptoethanol in a 250 mL centrifugation tube. The mixture was placed on ice for 1 h and then was centrifuged at 12,000× *g* for 15 min at 4 °C. The resulting supernatant (80 mL) was pipetted into a new tube, followed by addition of ammonia sulfate (from 0% to 40%) to precipitate proteins. The mixture was centrifuged at 12,000× *g* for 15 min at 4 °C and the resulting supernatant was disposed to a waste container. The remained residue was dissolved in 5 mL phosphate-buffer (0.1 M, pH 7.0) and centrifuged at 12,000× *g* for 10 min at 4 °C. The resulting supernatant containing crude proteins was used for ANR activity assay described below. 

Enzyme assay for crude enzyme extract was carried out by following a method reported by Xie et al. [21] and Zhang et al. [22] with slight modifications. In brief, enzyme assay was carried out in 15 mL capped polypropylene tubes. The reaction volume was 3.5 mL consisting of 100 mM phosphate buffer (pH 6.5), 2 mM NADPH, 0.5 mM substrate (cyanidin or delphinidin), and 300 μg crude enzyme extract. Boiled crude enzyme extract was used to replace crude enzyme in the reaction as one control. In addition, reactions without adding substrates were carried out as the other control. The enzymatic reactions were incubated for 40 min at 40 °C. All reactions were stopped by addition of 1 mL ethyl acetate (EA) to reaction tubes and vortexing, followed by centrifugation at 5000× *g* for 5 min. The resulting ethyl acetate supernatant phase was pipetted into a new tube. This step was repeated two times. Three times of EA extractions was pooled together to obtain 3 mL extract. EA was dried off via a rotary evaporation at room temperature. The remained residues were completely suspended in 100 μL HPLC-grade methanol, followed by centrifugation at 10,000× *g* for 2 min. The methanol phase was transferred to a new tube for HPLC analysis, which was as described above in the analysis of flavan-3-ols in leaves from field-grown Shuchazao plants. (−)-EC, (−)-Ca, (−)-GC, and (−)-EGC standards were used to identify metabolites from enzymatic reactions.

### 4.4. Isolation of Shuchazao ANR cDNA (CssANR) and Quantitative Reverse Transcription-Poly Chain Reaction (qRT-PCR) Analysis

Total RNA was isolated from mixed samples of buds and leaves (Figure 2a) using the RNAiso-mate for Plant Tissue Kit according to the manufacturer’s protocol (Takara, Tokyo, Japan). The first strand cDNA was synthesized using PrimeScript^®^ RT reagent Kit according to the manufacturer’s protocol (Takara, Tokyo, Japan). According to two cDNA sequences, KY615701.1 (CssANRa) and KY615702.1 (CssANRb), which we previously submitted to GenBank, the first pair of primers consisting of forward 5’-ATGGAAGCCCAACCGACA-3’ and reverse 5’-TCAATTCTTCAAAATCCC3’ was designed to amply for *CssANRa*. The second pair of primers consisting of forward 5’-ATGGCAATGGCAATGGCAACAAC-3’ and reverse 5’-TCAGTTCTGCAAAAGCCCCTTAG-3’ was designed to amplify *CssANRb*. The open reading frame (ORF) of *CssANRa* and *CssANRb* were amplified using a thermal gradient program that was composed of 5 min at 94 °C, 30 cycles of 30 s at 94 °C, 30 s at 62 °C, and 1 min at 72 °C, followed by a 10-min extension at 72 °C. The products of PCR were gel-purified using Takara MiniBEST Agarose Gel Extraction Kit (Takara) and ligated into the pMD18-T vector (Takara, Tokyo, Japan). The ligated products were transformed into *E. coli* strain DH5α competent cells using electroporation. Transformed cells were streaked on LB medium supplemented with 50 mg/L ampicillin. Positive colonies were selected to isolate recombinant plasmids. The resulting plasmids were termed pMD18-T-*CssANRa* and pMD18-T-*CssANRb* for sequencing at BGI (http://www.bgitechsolutions.com/).

In addition, primer pairs were designed for qRT-PCR analysis that was conducted using SYBR-Green PCR Master mix (Invitrogen, Shanghai, China) and were carried out on a LightCycler^®^ 480 System (Roche Diagnostics, Indianapolis, IN, USA). The primer pair for *CssANRa* included forward 5’-GAGTACTTCAAGGCTAAGGGG AT-3’ and reverse 5’-CAAGCAAACCAAGCAAAACC-3’. The primer pair for *CssANRb* included forward 5’-CTGGCAATCCAAGGAGTGC-3’ and 5’-GCCCCGTTCCATCAAGC-3’. The glyceraldehyde-3-phosphate dehydrogenase gene (*GAPDH*) is housekeeping gene in tea plants. It was used for normalization. The primer pair used for this gene consisted of forward 5’-TTGGCATCGTTGAGGGTCT-3’ and reverse 5’-CAGTGGGAACACGGAAAGC-3’. Values were normalized against the expression level of *GAPDH* [23]. Relative expression values were calculated with the 2^−△△Ct^ method. Values of gene expression were means of four replicates.

### 4.5. Analysis of ANR Sequences

Available *C. sinensis* ANR sequences curated at NCBI was identified and their amino acid sequences were aligned using ClustalX. In addition, four *C. sinensis* and 12 other species’ ANR amino acid sequences were used to construct a phylogenetic tree via Molecular Evolutionary Genetics Analysis version 5.0 (MEGA5.0; MegaSoftware, Tempe, AZ, USA) [24] using a neighbor–joining statistical method (with bootstrapping 1000 replicates) [25]. These 12 ANRs are MsANR (*Medicago sativa*, ADK95116.1, LcANR (*Lotus corniculatus*, ABC71333.1), FaANR (*Fragaria ananassa*, ABG76842.1), PaANR (*Prunus avium*, ADY15312.1), MdANR (*Malus domestica*, AEL79861.1), TcANR (*Theobroma cacao*, ADD51353.1), PtANR (*Populus trichocarpa*, XP_002305639.1), VvANR (*Vitis vinifera*, CAD91911), DkANR (*Diospyros kaki*, BAF56654.1), PcANR (*Pyrus communi*, ABB77695.1), GhANR (*Gossypium hirsutum*, ABM64802.1), TrANR (*Trifolium repens*, ADV31321.1). Four *C. sinensis* ANRs were CsANR1 (ADZ58168.1) and CsANR2 (ADZ58166.1) reported previously as well as CssANRa (ASU87432.1) and CssANRb (ASU87433.1) described here. The three-dimensional structures of CssANRa and CssANRb were modelled using homology modeling methods provided by Swiss Model (https://swissmodel.expasy.org/).

### 4.6. Induction of Recombinant CssANRa and CssANRb in E. coli

The pMD18-T-CssANRa and -CssANRb vectors containing the ORFs of *CssANRa* and *CssANRb* were digested using XbaI and SnaBI restriction enzymes. The resulting ORFs of *CssANRa* and *CssANRb* were separated on agarose gel using electrophoresis and then purified using the MiniBEST Plasmid Purification Kit (Takara) according to the manufacturer’s protocol. In addition, the pET31a^+^ vector was digested using the same restriction enzymes. Then, the ORFs of *CssANRa* and *CssANRb* were separately ligated to pET31a^+^ overnight using T4 DNA Ligase (Takara) under 15 °C. The ligation products were introduced to BL21 (DE3) competent cells using a common electroporation method. Transformed BL21 cells were streaked on agar-solidified medium containing 50 mg/L ampicillin. Antibiotic-resistant colonies were selected to confirm the presence of ORFs in the plasmids and positive colonies were used to induce protein expression. The new plasmids were named as pET31a^+^-*CssANRa* and -*CssANRb*, respectively. Furthermore, the pET31a^+^ vector was also introduced into BL21 cells as protein induction control. In addition to pET31a^+^, the ORF of *CssANRb* was ligated to the T7 SUMO vector (Invitrogen, K300-01) using the same ligation method. The ligation products were introduced to One Shot Mach1™-T1R Competent Cells (Invitrogen, K300-01) to select positive colonies on LB medium supplemented with 50 mg/L kanamycin. Kanamycin resistant colonies were selected to confirm the presence of *CssANRb*. The resulting new plasmid was named as T7 SUMO-*CssANRb*. In addition, T7 SUMO was introduced to the same competent cells as vector control for induction of recombinant protein expression. 

Five colonies each containing one of pET31a^+^-*CssANRa*, pET31a^+^-*CssANRb*, T7 SUMO-*CssANRb*, pET31a^+^, and T7 SUMO plasmids were used to induce protein expression. Each colony was inoculated into 10 mL LB broth containing 50 mg/L antibiotics (ampicillin for pET31a^+^-*CssANRa* and pET31a^+^ vector, and kanamycin for T7 SUMO-*CssANRb* and T7 SUMO vector) in an E-flask. All flasks were placed on a shake with a speed of 250 rpm at 37 °C. When cells were grown to a concentration indicated by an absorbent value about 0.6 that was recorded at 600 nm on an UV spectrophotometer, each flask was added isopropyl β-d-1-thiogalactopyranoside (IPTG) to a final concentration of 0.4 mM. Each flask was continuously shaken 3 h at 37 °C. Cells were harvested by centrifugation at 4000× *g* for 15 min at 4 °C. The remained cell pellet was suspended in 10 mL 100 mM phosphate buffer (pH 6.5) by vortexing. A T4 lyase was added to the suspension mixture with a final concentration of 100 µM. Then, the mixture was sonicated for 1 min on ice, followed by 20 min of centrifugation at 10,000× *g* at 4 °C. The supernatant was transferred to a new tube. Crude protein extracts were further treated using the MagnetHis Protein Purification System (Promega, Madison, WI, USA) to remove excessive proteins of *E. coli* according to the manufacturer’s protocol. Partially purified protein dissolved in 100 mM phosphate buffer (pH 6.5) was measured to estimate concentrations with the Bio-Rad Protein Assay system (Bio-RAD, California, CA, USA) and were stored in −80 °C until enzyme assay described below.

### 4.7. Enzyme Assays

Partially purified recombinant CssANRa and CssANRb (Figure 6a) were assayed using the method reported by Xie et al. [21] to optimize temperature and pH values. In all assays, experiments were carried out in 1 mL volume. Three replicates were completed for each reaction. The incubation time was 40 min. Boiled enzyme was used as control. In addition, reactions without addition of substrates were used as another control.

For optimization of temperature, enzyme reactions were carried out in 2 mL volume including 100 mM phosphate buffer (pH 6.5), 2 mM NADPH, 0.5 mM cyanidin, and 150 μg recombinant proteins. Tested temperature values included 0, 10, 20, 30, 40, 50 and 60 °C. 

To optimize pH value, enzyme reactions were performed in 2 mL volume consisting of a buffer, 2 mM NADPH, 0.5 mM cyanidin, and 150 μg recombinant CssANRa or CssANRb. Buffers tested included 100 mM citric acid phosphate buffer (pH 4.0~6.0), 100 mM phosphate buffer (pH 6.0~7.2), and 100 mM Tris-HCl buffer (pH 7.5~9.0) were prepared to obtain different pH values from 4.0 to 9.0. Tested pH values included 4.0. 4.5, 5.0, 5.5, 6.0, 6.5, 7.0, 7.5, 8.0 and 9.0. The incubation temperature was 40 °C.

Methods for stopping all reactions, extracting metabolites, and HPLC analysis were as described for crude enzyme assay.

### 4.8. Chiral Analysis of Products from Enzyme Reactions

HPLC using a normal-phase chiral column (catalog No. 80325; Chiral Technologies; 5 μm, 4.6 × 250 mm; Tokyo, Japan) was performed to determine the chirality of enzyme reaction products from cyanidin substrate according to a method reported by Pang et al. [17]. Elution buffer was composed of solvent A (hexane:acetic acid, 99.5:0.5, *v*/*v*,) and solvent B (ethanol:acetic acid, 99.5:0.5, *v*/*v*). Isomers were separated using a gradient program, which was composed of 0–20 min with 20% solvent B, 20–23 min with 20–50% B, 23–38 min with 50% B, and 38–40 min with 50–20% B. The flow rate was set at 1 mL/min. UV spectrum was recorded at 280 nm to characterize isomers. (−)-EC and (−)-Ca standards were used as control.

### 4.9. Construction of Binary Vectors, Tobacco Transformation, and Flower Sample Harvest

The Gateway^®^ Cloning System (Invitrogen, New York, NY, USA) was used to construct binary vectors according to a method reported by Lei et al. [26]. In brief, pairs of primers containing attB Gateway sequence were designed to amplify *CssANRa* and *CssANRb* to develop binary vectors. The primer pairs were CssANRa-attB (attB1 and attB2) and CssANRb-attB (attB1 and attB2) (Table S1). The pMD18-T-*CssANRa* and -*CssANRb* plasmids were used as template for PCR. The thermal program used for PCR was composed of 95 °C × 2 min, 35 cycles of 94 °C × 1 min, 56 °C × 1 min, and 72 °C × 1.5 min, and 72 °C for 10 min extension. The resulting PCR products were purified and then cloned to the entry vector pDONR207 with Gateway^®^ BP Clonase^®^ Enzyme mix according to the manufacturer’s instructions (Invitrogen). The resulting ligation products were introduced to competent DH5α cells, which were selected on agar-solidified LB plates containing gentamycin. Antibiotic-resistant colonies were selected and cultured to isolate recombinant entry vector. The positive entry vectors were incubated with the Gateway plant transformation destination vector pCB2004 using Gateway^®^ LR ClonaseTM enzyme (Invitrogen) according to the manufacturer’s protocol. The incubation mixtures were then introduced into competent DH5α cells, which were further selected on agar-solified LB medium containing 50 µg/mL kanamycin. Positive colonies were selected to isolate plasmids. Finally, two plasmids, namely pCB2004-*CssANRa* and -*CssANRb*, were created, in which two genes were controlled by a 35S promoter. These two vectors and empty pCB2004 (as control) were introduced into competent *Agrobacterium tumefaciens* strain EHA105 by electroporation, respectively. One positive colony was obtained for each vector. Positive colonies, namely EHA105-pCB2004-*CssANRa*, EHA105-pCB2004-*CssANRb*, EHA105-empty pCB2004, were selected on agar-solified LB medium containing 50 µg/mL kanamycin for genetic transformation.

A single colony, each for EHA105-pCB2004-*CssANRa*, EHA105-pCB2004-*CssANRb*, and EHA105-empty pCB2004, was inoculated into 20 mL liquid LB medium containing 50 mg/L kanamycin and 50 mg/L spectinomycin in 100 mL E-flask. Three flasks were placed on a rotary shaker at 200 rpm in the dark at 28 °C for overnight until OD value was about 0.6 at 600 nm. Cultured cells were transferred to a 50 mL sterile polypropylene centrifuge tube, followed by centrifugation at 4500× *g* for 10 min and disposal of supernatant. The remaining pellet was suspended in 20 mL liquid MS medium containing 100 μM acetosyringone (Sigma-Aldrich, St. Louis, MO, USA R40456), followed by centrifugation as above. This step was repeated once and the pellet was suspended in 20 mL liquid MS medium containing 100 μM acetosyringone to obtain activated cells for genetic transformation. 

Sterile seedlings of tobacco (*Nicotiana tabacum* ‘G28′) plants were grown on agar-solidified MS medium. Leaves of seedlings were cut into 1 × 1 cm discs. Infection of leaf discs with activated *A. tumefaciens* strain EHA105 described above and selection of transgenic plants on regeneration medium containing 25 mg/L phosphinothricin followed a protocol reported previously [27]. Gene specific primer pairs (CssANRa-test and CssANRb-test) were designed to screen transgenic plants (Table S1). RT-PCR as described above for expression profiling analysis was carried out to identify positive transgenic candidates. Positive transgenic vs. wild-type tobacco plants were planted into pot soil and then grown in a glass house, which was provided with natural light and maintained at 22–25 °C. Flower pigmentation was photographed every day. Flowers were collected from different developmental stages and then frozen in liquid nitrogen immediately. Frozen samples were stored at −80 °C until PA and anthocyanin analysis described below.

### 4.10. Analysis of PAs and Anthocyanins in Transgenic vs. Wild-Type Tobacco Flowers 

Flowers were ground into fine powder in liquid nitrogen to extract anthocyanins and PAs. Anthocyanins were extracted from 100 mg powdered tobacco flower samples as described previously [23]. Anthocyanin levels were estimated using absorbent values recorded at 530 nm on a UV spectrophotometer (HITACHI, Tokyo, Japan) [23]. PAs in flowers were extracted as reported previously [28]. The PA levels were determined using blue coloration via a reaction of PAs with 0.2% DMACA solution [0.2% (*w*/*v*) dissolved in methanol:concentrated HCl, 9:1]. After reaction for five min, samples were measured at 640 nm on a UV spectrophotometer (HITACHI, Tokyo, Japan) [17]. Absorbent values at 640 nm were used to compare PAs in transgenic vs. wild-type tobacco flower samples.

### 4.11. ANR Transcript Abundance Trend in Tissues 

Recently, eight transcriptomes were sequenced from buds, the 1st leaf, the 2nd leaf, young stem, old stem, and three developmental stage flower samples of two commercial verities, “Pingyangtezao (PYTZ)” and “Ruixue”. Transcriptomes of buds, the 1st leaf, the 2nd leaf, young stem, and old stem of PYTZ were sequenced by Gene Denovo Biotechnology Co. (Guangzhou, China). Although these transcriptomes have not been published, we were allowed to mine transcripts of tea *ANR* and transcription factor genes. Three developmental stages of flowers Ruixue were alabastrum (S1), half opened (bloom) flower (S2) and full bloom (S3). Transcriptomes from these flower stages were sequenced by the Beijing Genomics Institute (BGI, http://www.genomics.cn/, Shenzhen, China) [29]. Transcripts of total *ANR* cDNAs were extracted from these transcriptomes to compare its abundance in different tissues. Moreover, all unigenes in these transcriptomes were blasted against transcription factor (TF) database from plant (http://planttfdb.cbi.pku.edu.cn/, version 4.0) to annotate potential TFs. The resulting TF genes were further blasted against tree genomes (NCBI Sequence Read Archive Database No. PRJNA381277) [30] to unify sequences based on BLASTN 2.6.0+ (NCBI, U.S. National Library of Medicine, Bethesda, MD, USA) [31]. Transcripts of all TF genes were extracted from all eight transcriptomes and then analyzed using the omicshare cloud platform (http://www.omicshare.com/tools/Home/Soft/trend) to understand each TF gene expression trend pattern, those TF genes which with the same trend were grouped together to be tagged as one group. As a result, different profile groups were numerated into profile 1, 2, and so on.

## Supplementary Materials

The following are available online. Figure S1: Enzyme activity of recombinant CssANRa and CssANRb. Figure S2: Thirteen gene families and their member numbers obtained from annotation of 23 transcription factor genes that are coupled with the expression of *ANR* in buds, the 1st, the 2nd, young stem, and old stem. Figure S3: Twenty nine gene families and their member numbers obtained from annotation of 206 transcription factor genes that are coupled with expression of *ANR* in three developmental stages of flowers. Table S1: Primers used in the research.
