# Supplementary figures and images for: Metabolic Characterization of the Anthocyanidin Reductase Pathway Involved in the Biosynthesis of Flavan-3-ols in Elite Shuchazao Tea (Camellia sinensis) Cultivar in the Field
Source: Molecules. 2017 Dec 15;22(12):2241. doi: 10.3390/molecules22122241 (PMC6149802; doi:10.3390/molecules22122241)

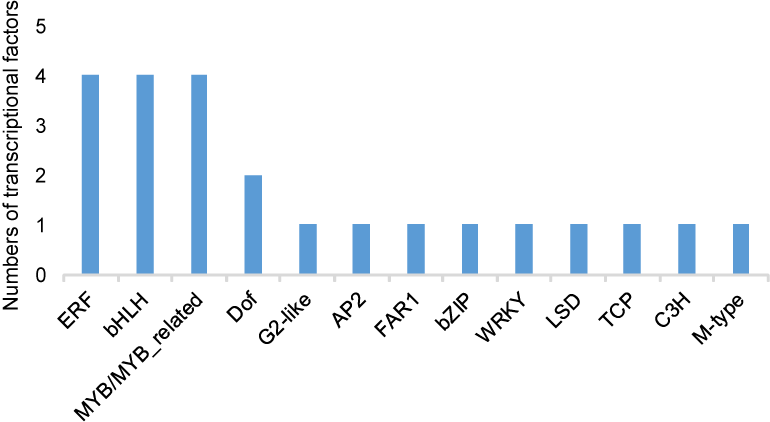

Supplement: Supplementary file 1 [file molecules-22-02241-s001.zip › FIG S/Fig S2.tif]

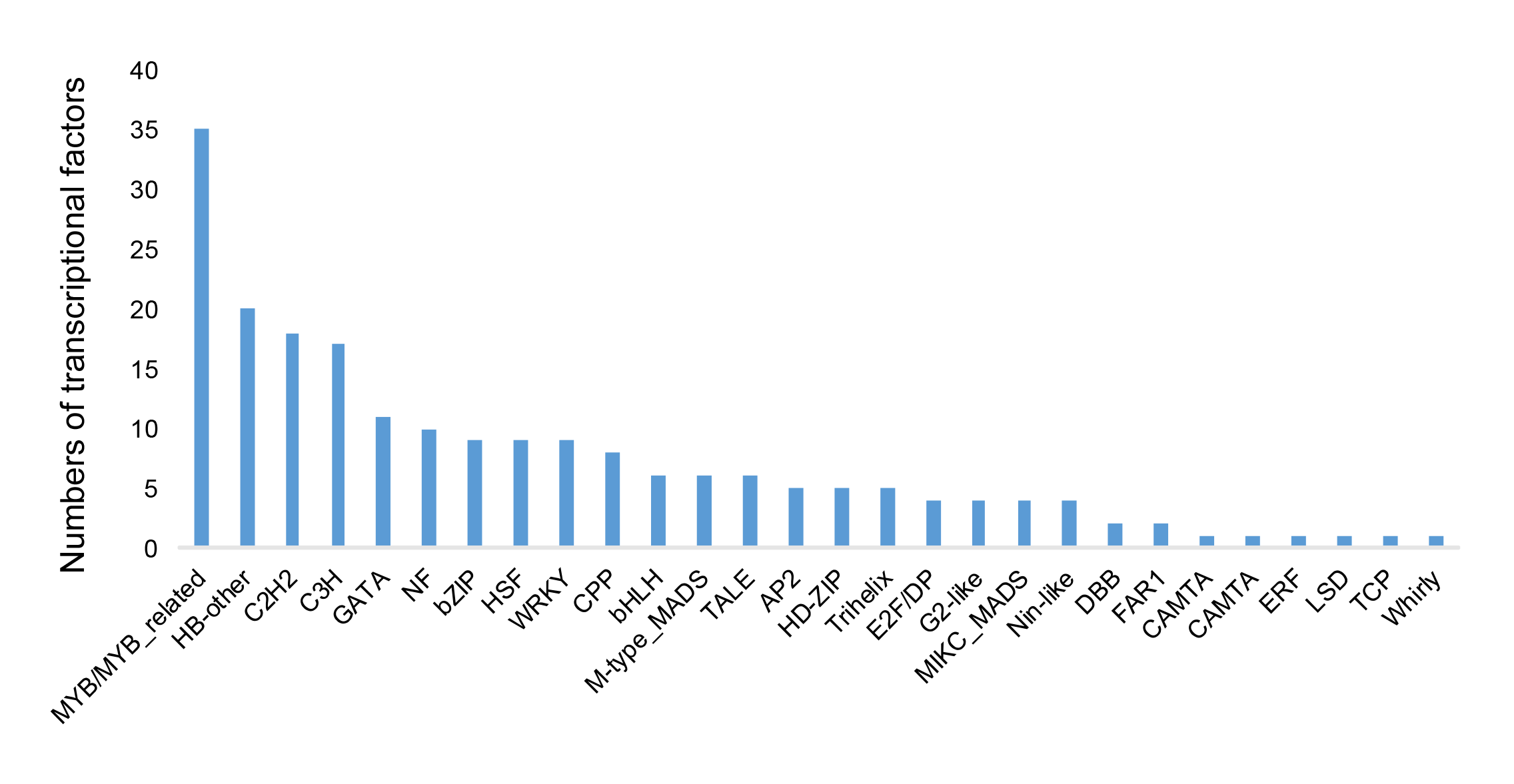

Supplement: Supplementary file 1 [file molecules-22-02241-s001.zip › FIG S/Fig S3.tif]
