# Supplementary material for: Metabolic Characterization of the Anthocyanidin Reductase Pathway Involved in the Biosynthesis of Flavan-3-ols in Elite Shuchazao Tea (Camellia sinensis) Cultivar in the Field
Source: Molecules. 2017 Dec 15;22(12):2241. doi: 10.3390/molecules22122241 (PMC6149802; doi:10.3390/molecules22122241)
Supplement: Supplementary file 1 [file molecules-22-02241-s001.zip › Table S.doc]

| Name of the primers | Sequences of the primers (5’-3’) |
| --- | --- |
| *CsANR*1-attB | attB1: GGGGACAAGTTTGTACAAAAAAGCAGGCTATGGAAGCCCAACCGACAGCTC |
|  | attB2: GGGGACCACTTTGTACAAGAAAGCTGGGTTCAATTCTTCAAAATCCCCTTAGCC |
| *CsANR*2-attB | attB1: GGGGACAAGTTTGTACAAAAAAGCAGGCTCCAATTCTTGCTGTGATCTCTCA |
|  | attB2: GGGGACCACTTTGTACAAGAAAGCTGGGTTGGTAGTGCCTTCAGTTCTGC |
| *CsANR1*-test | F: ATGGAAGCCCAACCGACA |
|  | R: TCAATTCTTCAAAATCC |
| *CsANR2*-test | F: ATGGCAATGGCAATGGCAAC |
|  | R: TCAGTTCTGCAAAAGCCCCT |

**Table S1 Primers used in the research**
